# Experimental Verification of the Possibility of Reducing Photoplethysmography Measurement Time for Stress Index Calculation

**DOI:** 10.3390/s23125511

**Published:** 2023-06-12

**Authors:** Seung-Gun Lee, Young Do Song, Eui Chul Lee

**Affiliations:** 1 Department of AI & Informatics, Graduate School, Sangmyung University, Hongjimun 2-Gil 20, Jongno-Gu, Seoul 03016, Republic of Korea; 202233053@sangmyung.kr (S.-G.L.); 202232031@sangmyung.kr (Y.D.S.); 2 Department of Human-Centered Artificial Intelligence, Sangmyung University, Hongjimun 2-Gil 20, Jongno-Gu, Seoul 03016, Republic of Korea

**Keywords:** stress index, ultra-short-term, pulse rate variability, machine learning, regressor

## Abstract

Stress is a direct or indirect cause of reduced work efficiency in daily life. It can damage physical and mental health, leading to cardiovascular disease and depression. With increased interest and awareness of the risks of stress in modern society, there is a growing demand for quick assessment and monitoring of stress levels. Traditional ultra-short-term stress measurement classifies stress situations using heart rate variability (HRV) or pulse rate variability (PRV) information extracted from electrocardiogram (ECG) or photoplethysmography (PPG) signals. However, it requires more than one minute, making it difficult to monitor stress status in real-time and accurately predict stress levels. In this paper, stress indices were predicted using PRV indices acquired at different lengths of time (60 s, 50 s, 40 s, 30 s, 20 s, 10 s, and 5 s) for the purpose of real-time stress monitoring. Stress was predicted with Extra Tree Regressor, Random Forest Regressor, and Gradient Boost Regressor models using a valid PRV index for each data acquisition time. The predicted stress index was evaluated using an $R^{2}$ score between the predicted stress index and the actual stress index calculated from one minute of the PPG signal. The average $R^{2}$ score of the three models by the data acquisition time was 0.2194 at 5 s, 0.7600 at 10 s, 0.8846 at 20 s, 0.9263 at 30 s, 0.9501 at 40 s, 0.9733 at 50 s, and 0.9909 at 60 s. Thus, when stress was predicted using PPG data acquired for 10 s or more, the $R^{2}$ score was confirmed to be over 0.7.

## 1. Introduction

Stress is a significant issue in today’s society. According to the World Health Organization (WHO), stress-related productivity loss causes more than $1 trillion in damages annually, and 15% of adult workers suffer from mental disorders [1]. Long-term stress undermines physical and mental health, leading to cardiovascular disease, diabetes, depression, and other illnesses [2,3]. Therefore, it is crucial to recognize and manage stress for the sake of physical and mental well-being.

In the past, stress was assessed through questionnaires [4]. However, wearable devices have been used recently to monitor stress by measuring biological signals. With recent advancements in wearable technology, there has been an increase in interest and demand for healthcare that can monitor stress status in real time. The most commonly used biological signals in wearable devices are photoplethysmography (PPG) and the electrocardiogram (ECG) [5,6,7,8], which measure the blood volume pulse (BVP) signal from a heartbeat. Stress assessment using an ECG is typically explained by analyzing the heart rate variability (HRV). HRV represents a state of the autonomic nervous system by analyzing the R-R interval (RRI), which is the distance between peaks of blood volume pulse (BVP) signals obtained from the ECG.

Stress is characterized by excessive activation of the sympathetic nervous system in the autonomic nervous system [9]. HRV is widely used and the most accurate quantitative physiological measurement of stress. Previous studies have used HRV indicators to recognize stress situations [10,11,12]. However, HRV analysis requires a lot of electrode placement and sophisticated measurement equipment, which can cause user discomfort. In addition, for short-term HRV analysis, which is often used for stress measurement, the European Society of Cardiology and the North American Society of Pacing and Electrophysiology Task Force recommend a minimum of 5 min of HRV measurement [13,14,15,16], making it difficult to evaluate and monitor an individual’s stress state in real-time.

PPG can be used similarly to an ECG for HRV measurement. Studies have shown that PPG is better for detecting mental stress than an ECG [17]. The micro-variability of peak-to-peak interval (PPI) detected from the BVP signal of PPG, called the pulse rate variability (PRV), can indicate the state of the autonomic nervous system, which is associated with the interaction between the sympathetic and parasympathetic nervous systems [18]. Furthermore, it has been proven that features extracted from the HRV and PRV are highly consistent. Thus, PRV can be used as an alternative to HRV for stress evaluation [19]. As such, many studies use physiological signals for stress detection. In addition, as demand for real-time stress state monitoring increases, many studies have investigated ways to perform ultra-short-term stress detection based on heart rate variabilities such as PRV and HRV [20,21].

For example, Castaldo et al. [22] have proposed a method for identifying stress based on a 3-min HRV analysis, unlike the conventional approach of analyzing mental stress in the long- and short-term. Jiao et al. [23] have validated the feasibility of ultra-short-term PRV for stress assessment, achieving accuracies of 90.77%, 95.26%, and 95.26% with data lengths of 1 min, 3 min, and 5 min, respectively, using a machine learning classification model. This provides a foundation for real-time monitoring and analysis-based mental stress classification technology. Castaldo et al. [24] have confirmed the consistency of six HRV features (Mean NNI, Std NNI, Mean HR, Std HR, HF, and SD2) across different time lengths from 5 min to 1 min. In the HRV features used, NNI is the normal-to-normal interval, HR is the heart rate, and HF is high frequency. They demonstrated high performance in stress classification using ultra-short-term HRV features, showing the ability to use the HRV features detected for more than one minute to automatically detect mental stress. Purnamasari et al. [25] have conducted a study to classify stress situations using an eight-feature k-Nearest Neighbor (k-NN) algorithm based on HRV data measured during stress and relaxation situations for one minute.

Although existing ultra-short stress assessment techniques have reduced the time required from the previous 5 min, they still cannot monitor stress status in real time. Furthermore, because they simply classify stress situations, there is a lack of quantitative evaluation information on current stress. Therefore, in this paper, we predicted a stress index for the next minute using the PRV detected from PPG. The Baevsky Stress Index [26] was used for the one-minute stress index, and the PRV index was used for stress index prediction. We compared 5-min PRV indicators with PRV indicators for 60, 50, 40, 30, 20, 10, and 5 s and used only correlated indicators to increase the reliability and reduce the stress measurement time.

## 2. Methods

### 2.1. Experimental Design

The PPG sensor of a Ubpulse 360 (LAXTHA, Daejeon, Republic of Korea) device used in the experiment for BVP measurement had a sampling rate of 255 [27]. PRV analysis and stress index calculations were performed using the PPI of the BVP signal detected from the PPG. Stress-related studies have found that short-term intense exercise can activate the sympathetic nervous system and increase stress [28]. To determine the level of stress, BVP signals were collected in a resting state and after exercise. BVP signals were collected from participants in a relaxed sitting position, after two sets of push-ups and after four sets of push-ups. One set of push-ups consisted of ten repetitions. In the resting task, BVP signals were collected with a PPG sensor attached to the finger of the seated participant, and BVP signals after each exercise task were collected while the participant was sitting down after the exercise was completed. A total of 17 participants took part in the experiment. Participants were recruited through campus advertising. The participants consisted of 8 men and 9 women, with an average age of 25. The PPG sensor collected BVP signals for six minutes, and participants were asked to minimize movement during the collection of BVP signals.

### 2.2. Preprocessing

**Figure 1:**
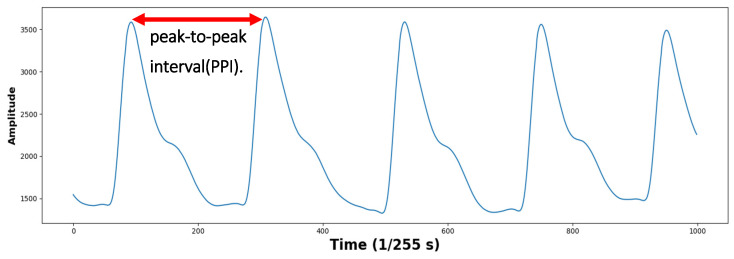
Example of photoplethysmography signal and peak-to-peak intervals (PPIs).

**Figure 2:**
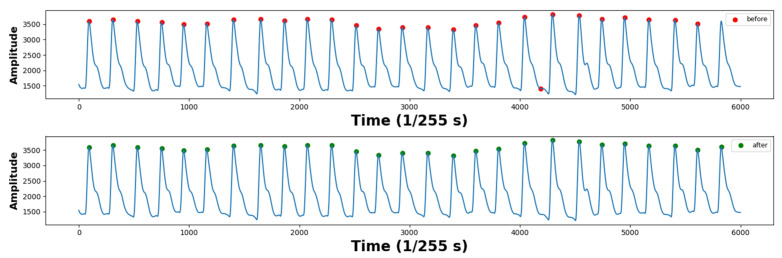
Example of peak detection before and after preprocessing.

**Figure 3:**
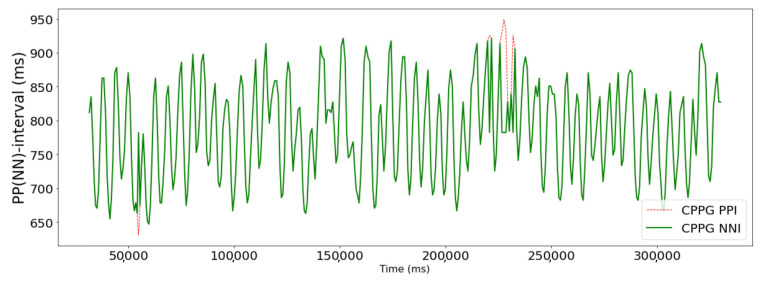
Example of normal-to-normal interval (NNI) as a preprocessing result of peak-to-peak interval (PPI).

When measuring BVP signals using a PPG measuring device, a lot of noise is distributed in the signal at the beginning and end points. Therefore, signals collected from the first 30 s and the last 30 s were discarded to obtain 5 min of data. As a result, 51 PPG signals were generated. The average heart rate of a person is around 60–100 beats per minute. It can increase up to 180 beats per minute during exercise [29]. It was confirmed that the intensity of exercise and heart rate increased proportionally. The maximum heart rate recorded was 158 beats per minute. To remove noise in the signal, band-pass filtering was performed, only leaving the 0.5 Hz~3.0 Hz component corresponding to the heart rate [30,31]. The PPI was extracted by detecting peaks after filtering. The time unit of the PPI was in milliseconds (ms). The PPG waveform and PPI examples are shown in Figure 1. To remove the PPI that deviated from the average distribution, the PPI with a z-score value greater than or equal to the threshold value, T, was removed, and T was empirically set to 2. Figure 2 shows an example of a PPG signal with abnormal peaks removed. To calculate the NNI, the removed PPI was replaced with the median value between the previous PPI and the subsequent PPI. An example of this is shown in Figure 3.

### 2.3. PRV and Stress

To use the PRV parameter for stress prediction, PRV was extracted from the NNI obtained through previous preprocessing steps. The time unit of the NNI was in milliseconds (ms). Only the time domain features of PRV were extracted and analyzed. Extracted time domain features [32] are shown in Table 1.

**Figure 4:**
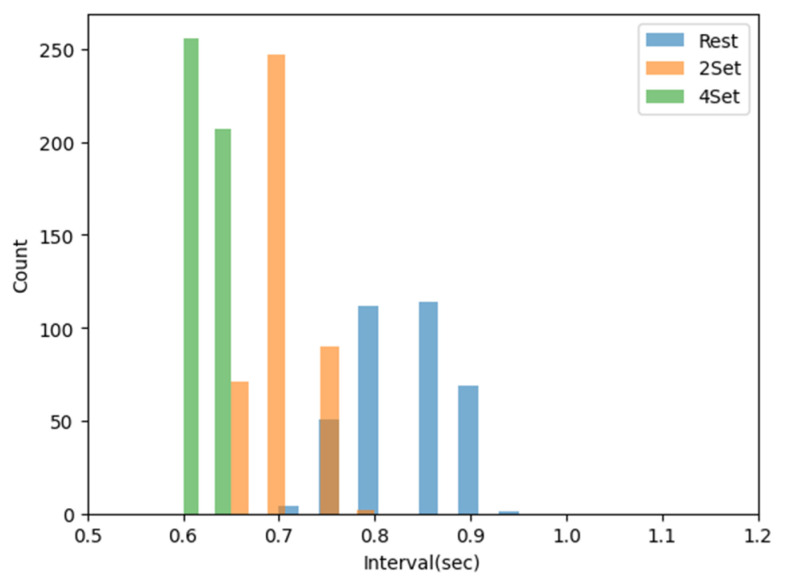
Example of normal-to-normal interval (NNI) histograms before and after exercise.

The stress index was calculated using the Baevsky Stress Index [33]. The Baevsky Stress Index (SI) was calculated using the following Equation (1):

(1) $$SI=\frac{AM_{o}\times 100\%}{2M_{o}\times M_{x}DM_{n}}$$

where mode ($M_{o}$) was the most frequent NNI expressed in seconds, and the amplitude of mode ($AM_{o}$) was the percentage of the total measured NNI that included the $M_{o}$ in the bin. It was calculated using NNIs with a bin width of 50 ms. The variation range ($M_{x}DM_{n}$) was the difference in seconds between the longest NNI $\left(M_{x}\right)$ and the shortest NNI ($M_{n}$), indicating the degree of variability in the interval. The Baevsky Stress Index (SI) was calculated using Equation (1). It reflected stress levels and activity of the autonomic nervous system. When the sympathetic nervous system is activated, the duration range of intervals decreases, and an increase in the number of intervals with similar durations is reflected as an increase in the $AM_{o}$, representing stress. As the $AM_{o}$ increases, the histogram becomes narrower and higher, indicating an increase in stress. The normal range of the SI is 80–150. Mild stress increases the SI by 1.5–2 times, while severe stress increases the SI by 5–10 times [33]. In [34], activation of the sympathetic nervous system was calculated using the SI. The SI was proven to be related to the actual activation of the sympathetic nervous system. In [35], a driver’s stress was evaluated using the SI. It was mentioned that an increase in the SI reflected an increase in stress, such as vessel contraction. Therefore, the SI was calculated using the previously obtained NNI to evaluate stress. Figure 4 shows a histogram of the NNI distribution in rest and exercise states. In this study, the square root of the SI calculated using Equation (1) was used to minimize the influence of outliers [34,36].

### 2.4. Feature Selection and Datasets

**Figure 5:**
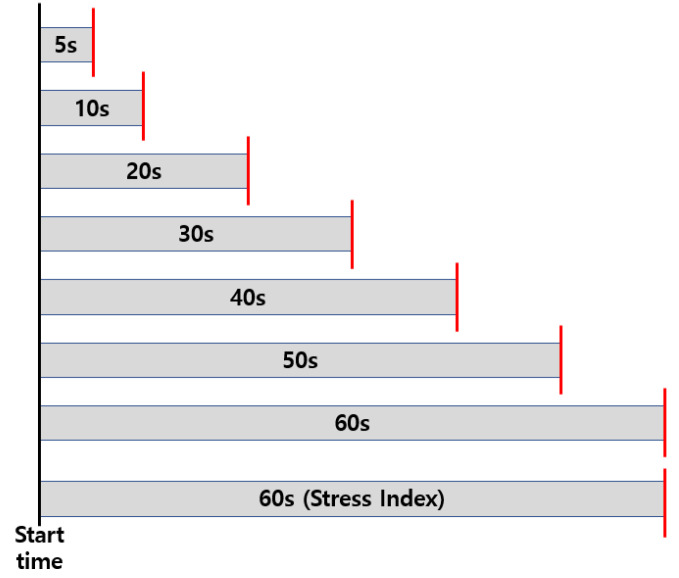
A method for creating inputs and labels for a regression model from the acquired 5-min photoplethysmography data.

In this study, we investigated the minimum required duration for predicting the stress index using photoplethysmography (PPG) signals by dividing a 5-min PPG signal into intervals of 60 s, 50 s, 40 s, 30 s, 20 s, 10 s, and 5 s. To increase the amount of data, we extracted time domain features of pulse rate variability (PRV) from the PPG signal by sliding a window every 1 s. We only used features that had an $R^{2}$ score of 0.6 or higher between the PRV time domain feature calculated from the recommended 5-min length PPG signal [13,14,15,16] and the PRV time domain feature calculated from PPG data of different lengths to predict the stress index from PPG data. Table 2 shows the $R^{2}$ score between the selected PRV feature for each data collection time of 5 to 60 s and the PRV feature calculated from the 5-min data. The $R^{2}$ score specified in the Table 2 is displayed in blue if it is greater than or equal to 0.6 and in red if it is less than 0.6. We found that as the measurement time increased, the number of features with high correlation increased, and the number of features with an $R^{2}$ score of 0.6 or more was 5 at 5 and 10 s, 8 at 20 and 30 s, and 9 at 40, 50 and 60 s, respectively, for each data collection time (a correlation graph for each $R^{2}$ score can be found in Appendix A). Due to the different scales of each selected feature, we used a normalizer as a scaling method to configure the shape used as the input to the regression model. Additionally, to predict the stress index, we constructed a dataset using the PRV time domain feature calculated by the PPG data acquisition time as the input data and the square root of the SI calculated from the PPG signal obtained for 1 min after the start of PPG acquisition as the label, as shown in Figure 5 [37,38]. As a result, the label is the square root of the SI derived from the PPG signal collected for 1 min from the PPG measurement time, and the input feature used is the HRV time domain index derived from the measurement time.

### 2.5. Regressor Model

Models used to predict the stress index in this paper included the Extra Tree Regressor [39], Gradient Boosting Regressor [40], K-Neighbor Regressor [41], and Linear Regressor [42]. The Extra Tree Regressor and Gradient Boosting Regressor are ensemble learning methods that can prevent overfitting, reduce local minima, and increase interpretability of the model [43]. The Extra Tree Regressor combines multiple decision trees by randomly selecting splitting points, allowing each tree to learn independently. This makes the decision boundary of the model diverse and improves its generalization performance. To account for large individual differences in stress levels, a model with randomness was chosen. The Gradient Boosting Regressor learns the model by calculating residuals (i.e., differences between the actual error and the predicted error) and minimizing residuals. The new model that minimizes the residual was added to the previous model. Ultimately, the final prediction is made by combining the predictions of all models. The K-Neighbor Regressor is one of the supervised learning algorithms that can find the k-closest neighbors of a specific point, calculate their average value, and derive the prediction value. It learns the relationship between input and output values by measuring the distance between each data point and predicting the closest neighbor. The Linear Regressor is a regression technique that models the linear correlation between one or more input data and the output value. It typically uses the least squares method [44] to learn the model. It models the relationship between the input data and output value as a linear equation. It predicts the output value by assigning a weight to each feature.

## 3. Results

**Figure 6:**
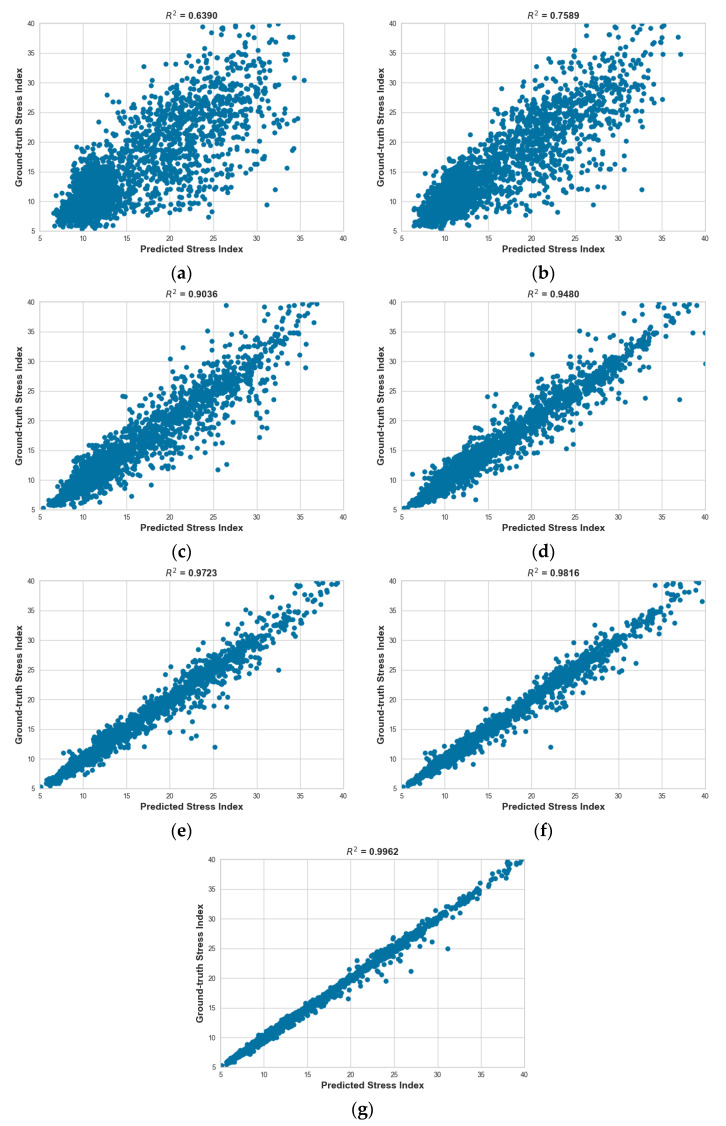
Graph between predicted stress index and ground-truth stress index at Extra Tree Regressor. (**a**) 5 s; (**b**) 10 s; (**c**) 20 s; (**d**) 30 s; (**e**) 40 s; (**f**) 50 s; (**g**) 60 s.

Stress index prediction results were analyzed using a regression model based on the PRV extracted from PPG data with lengths of 60 s, 50 s, 40 s, 30 s, 20 s, 10 s, and 5 s. The Extra Tree Regressor, Gradient Boosting Regressor, K-Nearest Neighbor Regressor, and Linear Regression were used to predict the stress index. The evaluation metric for the predicted stress index was the $R^{2}$ score calculated using the square root of the Baevsky Stress Index, which was obtained from the PPG signal measured for one minute. The $R^{2}$ score was used to numerically represent the regression accuracy for the actual stress index. $R^{2}$ scores for the Extra Tree Regressor using PPG signals of 5, 10, 20, 30, 40, 50, and 60 s to predict the stress index were 0.6390, 0.7589, 0.9036, 0.9480, 0.9723, 0.9816, and 0.9962, respectively. $R^{2}$ scores for the Gradient Boosting Regressor using PPG signals of 5, 10, 20, 30, 40, 50, and 60 s to predict the stress index were 0.6892, 0.7606, 0.8484, 0.8905, 0.9290, 0.9600, and 0.9850, respectively. Those for the K-Nearest Neighbor Regressor using PPG signals of 5, 10, 20, 30, 40, 50, and 60 s to predict the stress index were 0.6284, 0.7403, 0.8783, 0.9286, 0.9635, 0.9759, and 0.9933, respectively. For the Linear Regression, $R^{2}$ scores using PPG signals of 5, 10, 20, 30, 40, 50, and 60 s to predict the stress index were 0.6305, 0.7056, 0.7740, 0.8189, 0.8705, 0.9172, and 0.9475, respectively. These $R^{2}$ score results for each model are presented as average values of the $R^{2}$ scores from a 10-fold cross-validation based on the four regressors used in this study. They were rounded to four decimal places in Table 3. The $R^{2}$ score averages for the four regressors based on PPG data collection times of 5, 10, 20, 30, 40, 50, and 60 s were 0.6467, 0.7414, 0.8511, 0.8965, 0.9338, 0.9586, and 0.9805, respectively. It was found that the longer the data acquisition time to predict the outcome of the four models, the higher the $R^{2}$ score. It was confirmed that the data acquisition time was 10 s or more when the $R^{2}$ score was 0.7 or higher. Among the results of this paper, the graph of the predicted stress index and the actual stress index of the Extra Tree Regressor model is shown in Figure 6 (graphs of the predicted and actual stress indices for the remaining Gradient Boosting Regressor, K-Nearest Neighbor Regressor, and Linear Regression models can be found in Appendix B). The x-axis of the graph represents the predicted stress level, and the y-axis represents the actual stress level measured over 1 min. The results on the graph provide a visual representation of the $R^{2}$ score increasing as the data acquisition time increases.

## 4. Conclusions

This study differs from previous research on classifying stress situations in that it allows for the quantitative evaluation of stress by numerically observing current stress levels. Using a regression model, stress levels were predicted for one minute after the acquisition start time based on PPG data collected at intervals of 5 to 60 s while shortening the time. Features used for stress prediction were selected based on PRV indicators from 5 min of acquired PPG data and the $R^{2}$ scores of each PRV indicator calculated from the PPG data ranging from 5 to 60 s. Only features with $R^{2}$ scores of 0.6 or higher were used to increase reliability. It was confirmed that the number of features with $R^{2}$ scores of 0.6 or higher increased as the measurement time increased. Ultimately, for a 60-s measurement, the nine possible features with $R^{2}$ scores of 0.6 or higher were the Mean NNI, SDNN, SDSD, RMSSD, Median NNI, CVSD, Mean HR, MIN HR, and Max NNI. It was confirmed that the PRV indicators changed with an increase in heart rate when experiencing actual stress. PRV indicators also changed with respect to the measurement time. The performance of the regression model was validated for each time point using $R^{2}$ scores. The regression models used were the Extra Tree Regressor, Gradient Boosting Regressor, K-Nearest Neighbors Regressor, and Linear Regression. The stress prediction results were verified using 10-fold cross-validation. It was observed that the performances of the regression models based on decision trees were generally better than that of the Linear Regression for stress prediction. The $R^{2}$ scores of the predicted stress levels for each time point were above 0.7 for measurement times of over 10 s. As the measurement time increased, the $R^{2}$ score also increased. The highest $R^{2}$ score between predicted and actual stress levels at 60 s was 0.9962 using the Extra Tree Regressor. This study predicted a stress index for the following minutes using PRV indices calculated from PPG signals acquired every 5 s, starting from 60 s. Four regression models were used for each time period to compare and validate the predicted stress index with the actual stress index through a 10-fold cross-validation. Significant results were obtained using PRV indices calculated from PPG signals longer than 10 s, with an $R^{2}$ score of 0.7 or higher, indicating that even short PPG signals of 10 s could provide meaningful results for estimating stress.

In future studies, we plan to predict the stress index non-invasively using RPPG signals. Since RPPG signals are vulnerable to noise, such as ambient light and motion artifacts, we plan to investigate RPPG signals that are robust against noise and use them to predict stress.

## Figures and Tables

**Table 1:** Pulse rate variability of time domain features.

Parameter	Description
Mean NNI	The mean of RR intervals
SDNN	The standard deviation of the time interval between successive normal heartbeats
SDSD	The standard deviation of differences between adjacent RR intervals
RMSSD	The square root of the mean of the sum of the squares of differences between adjacent NN intervals
Median NNI	Median absolute values of the successive differences between the RR intervals
NNI 50	Number of interval differences of successive RR intervals greater than 50 ms
pNNI 50	The proportion derived by dividing NNI 50 (the number of interval differences of successive RR intervals greater than 50 ms) by the total number of RR intervals
NNI 20	Number of interval differences of successive RR intervals greater than 20 ms
pNNI 20	The proportion derived by dividing NNI 20 (the number of interval differences of successive RR intervals greater than 20 ms) by the total number of RR intervals
Range NNI	Difference between the maximum and minimum NN intervals
CVSD	Coefficient of variation of successive differences equal to the RMSSD divided by Mean NNI
CVNNI	Coefficient of variation equal to the ratio of SDNN divided by Mean NNI
Mean HR	The mean heart rate
Max HR	Max heart rate
Min HR	Min heart rate
Std HR	Standard deviation of heart rate
Max NNI	The max of RR intervals
Min NNI	The min of RR intervals

**Table 2:** $R^{2}$ scores between PRV index for 5 min and PRV index for 5 to 60 s.

	Mean NNI	SDNN	SDSD	RMSSD	Median NNI	CVSD	Mean HR	Min HR	Max NNI
5 s	0.9096	−0.0089	0.3678	0.5448	0.8963	−0.2537	0.9100	0.6410	0.6301
10 s	0.9350	0.2859	0.5741	0.5848	0.9272	0.5637	0.9363	0.7518	0.7576
20 s	0.9516	0.4887	0.8060	0.8074	0.9476	0.7174	0.9521	0.8250	0.8387
30 s	0.9606	0.5890	0.8468	0.8473	0.9582	0.7731	0.9610	0.8587	0.8731
40 s	0.9674	0.6472	0.8616	0.8619	0.9649	0.7823	0.9676	0.8798	0.8934
50 s	0.9723	0.6940	0.8623	0.8624	0.9705	0.7878	0.9722	0.8956	0.9080
60 s	0.9759	0.7330	0.8830	0.8831	0.9745	0.8081	0.9755	0.9079	0.9187

Features used for prediction: marked in blue, features not used for prediction: marked in red.

**Table 3:** $R^{2}$ scores of stress prediction results of Extra Tree Regressor at 5 to 60 s.

	5 s	10 s	20 s	30 s	40 s	50 s	60 s
Extra Tree Regressor	0.6390	0.7589	0.9036	0.9480	0.9723	0.9816	0.9962
Gradient Boosting Regressor	0.6892	0.7606	0.8484	0.8905	0.9290	0.9600	0.9850
K-Nearest Neighbor Regressor	0.6284	0.7403	0.8783	0.9286	0.9635	0.9759	0.9933
Linear Regression	0.6305	0.7056	0.7740	0.8189	0.8705	0.9172	0.9475
Mean	0.6467	0.7414	0.8511	0.8965	0.9338	0.9586	0.9805

**Figure A1:**
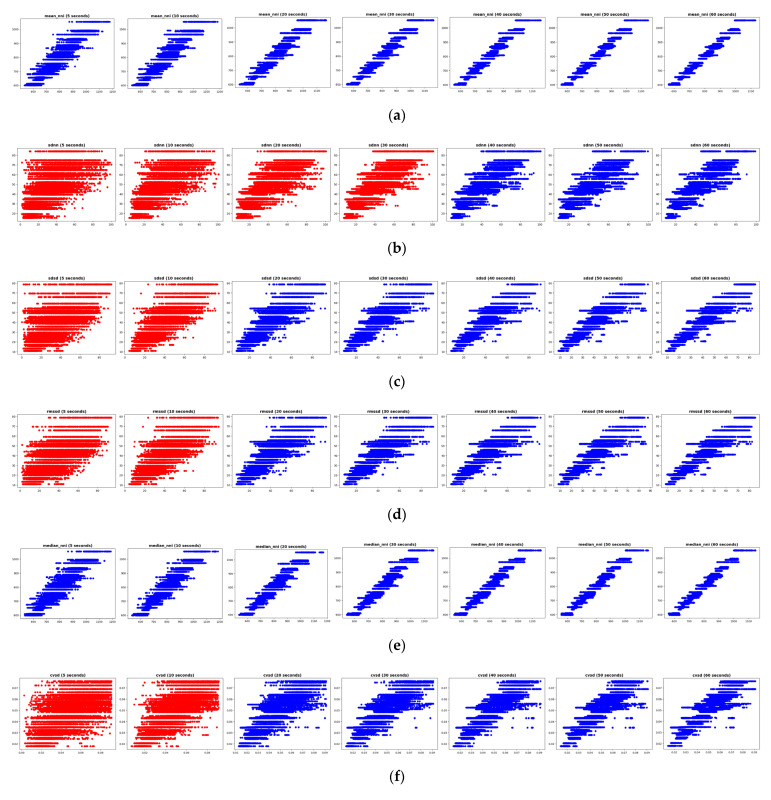

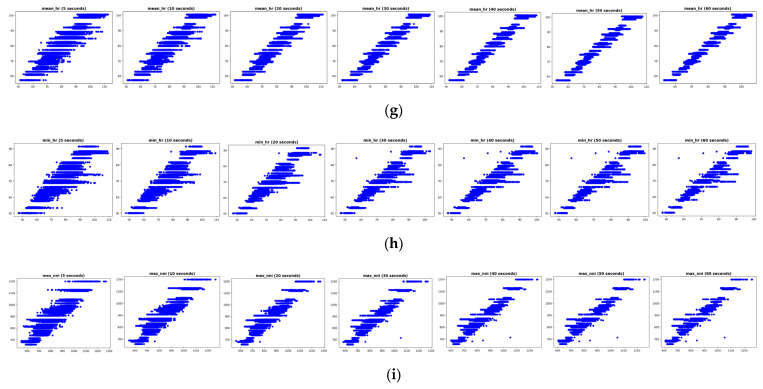
A graph showing the correlation between selected PRV features and 5-min PRV. (**a**) graph of Mean NNI by acquisition time; (**b**) graph of SDNN by acquisition time; (**c**) graph of SDSD by acquisition time; (**d**) graph of RMSSD by acquisition time; (**e**) graph of Median NNI by acquisition time; (**f**) graph of CVSD by acquisition time; (**g**) graph of Mean HR by acquisition time; (**h**) graph of Min HR by acquisition time; (**i**) graph of Max NNI by acquisition time.

**Figure A2:**
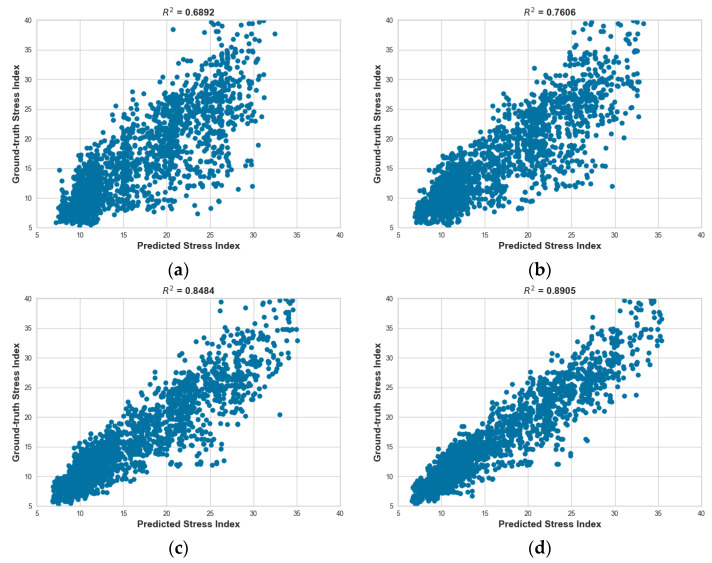

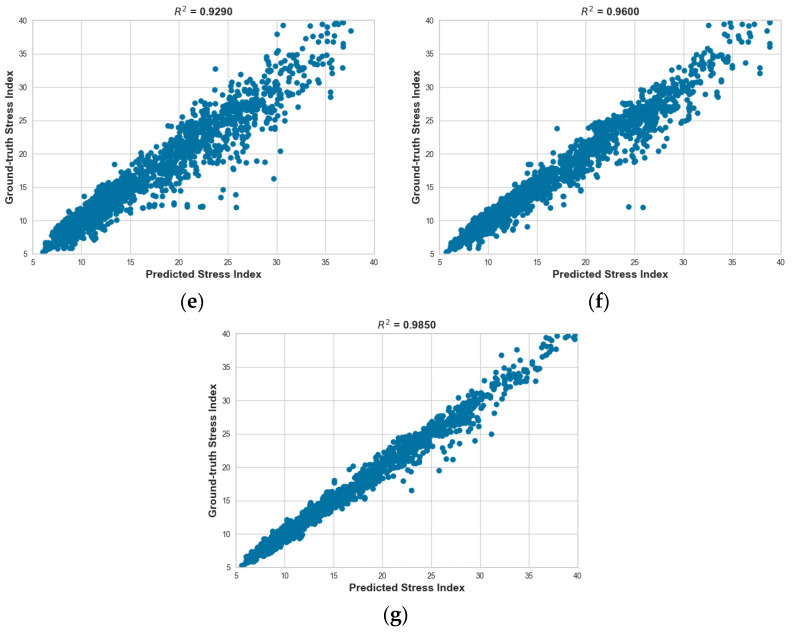
Graph between predicted stress index and ground-truth stress index at Gradient Boosting Regressor. (**a**) 5 s; (**b**) 10 s; (**c**) 20 s; (**d**) 30 s; (**e**) 40 s; (**f**) 50 s; (**g**) 60 s.

**Figure A3:**
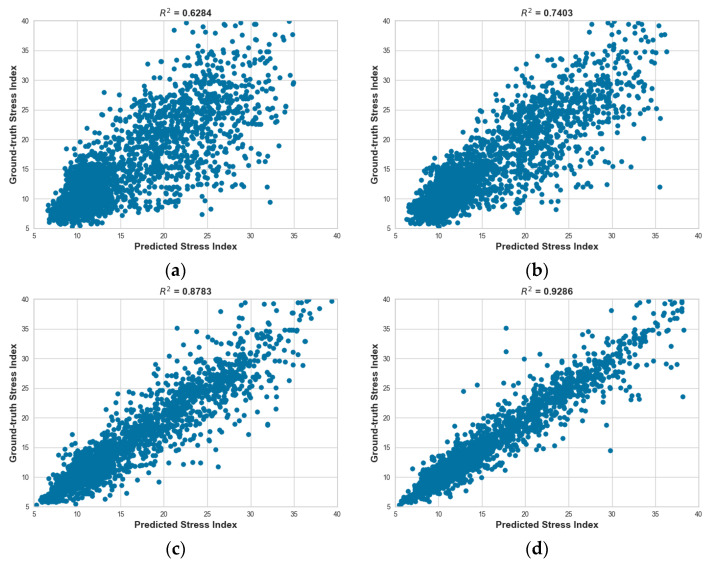

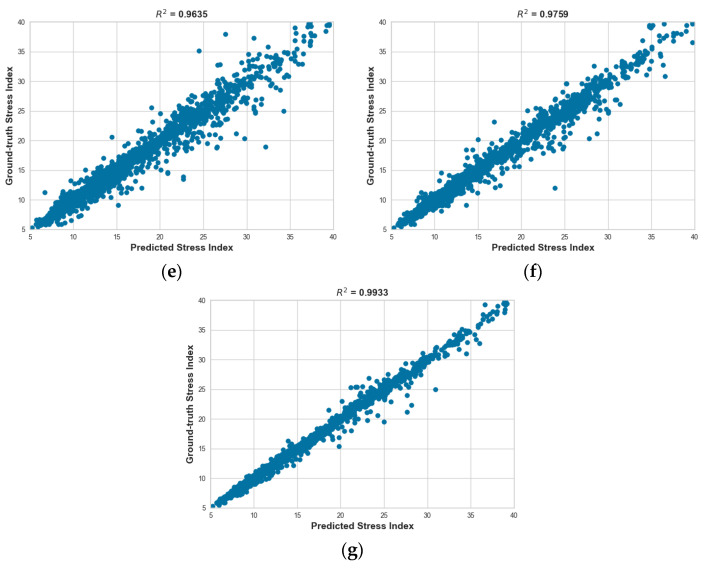
Graph between predicted stress index and ground-truth stress index at K-Nearest Neighbor Regressor. (**a**) 5 s; (**b**) 10 s; (**c**) 20 s; (**d**) 30 s; (**e**) 40 s; (**f**) 50 s; (**g**) 60 s.

**Figure A4:**
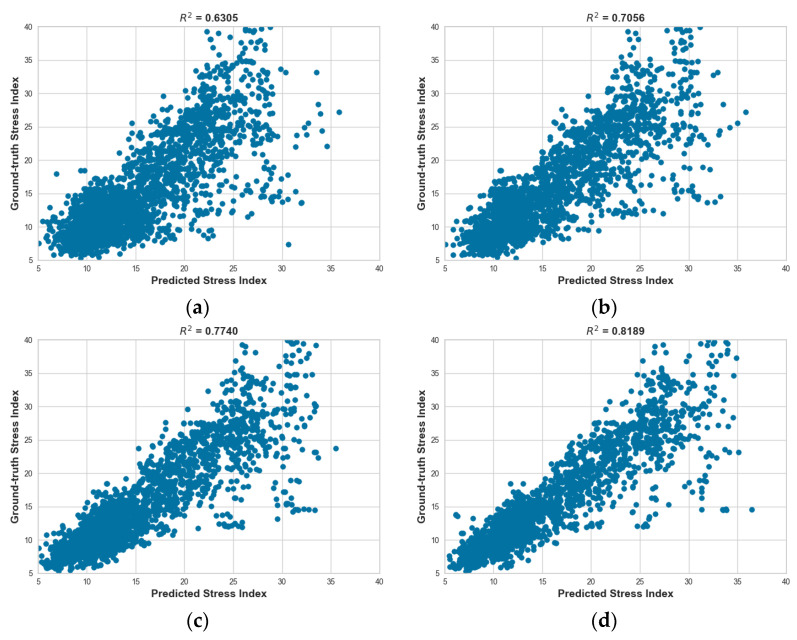

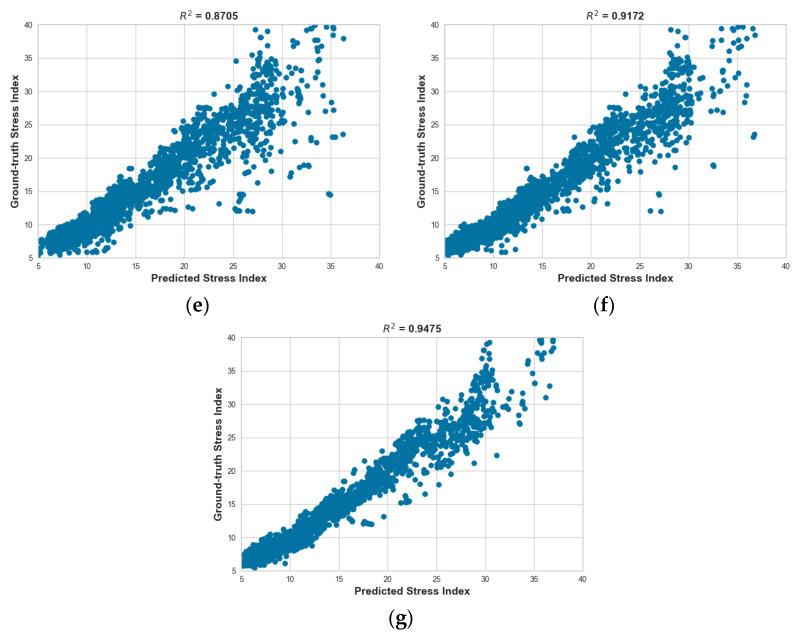
Graph between predicted stress index and ground-truth stress index at Linear Regression. (**a**) 5 s; (**b**) 10 se; (**c**) 20 s; (**d**) 30 s; (**e**) 40 s; (**f**) 50 s; (**g**) 60 s.

## Data Availability

The obtained data cannot be shared because it was agreed that it could be used only for this study.

## Appendix A. Correlation Graph between PRV Metrics by Data Acquisition Time and 5-Min PRV Metrics

In this section, we present a graph visualizing the correlation between nine PRV time domain features that are significant for predicting the stress index at 5 s, 10 s, 20 s, 30 s, 40 s, 50 s, and 60 s, and each PRV time domain feature at 5 min (Figure A1). The y-axis of the graph represents the PRV index of PPG obtained over 5 min, and the x-axis represents the PRV index of PPG obtained at each data collection time. Among the features analyzed for correlation, if the $R^{2}$ score was less than 0.6, the correlation graph of the feature that was not used was red, and for the feature that was used if the $R^{2}$ score was 0.6 or more, the graph was used in blue.

## Appendix B. Correlation Graph of the Model’s Stress Prediction Results

In this section, correlation graphs of predicted stress indices and actual stress indices of three models, the Gradient Boosting Regressor (Figure A2), K-Nearest Neighbor Regressor (Figure A3), and Linear Regression (Figure A4), are shown.
